# Particle counting immunoassay (PACIA) V- its application to the determination of human placental lactogen

**DOI:** 10.1155/S1463924680000442

**Published:** 1980

**Authors:** A. E. Leek, F. de Steenwinkel, C. L. Cambiaso, P. L. Masson

**Affiliations:** 1 Technia Diagnostics Ltd. City Road London ECVIJX UK; 2 St. Antonius hove Burgermeester Banninglaan 1 Leidschendam The Netherlands; 3 Unit of Experimental Medicine International Institute of Cellular and Molecular Pathology Université Catholique de Louvain Brussels B–1200 Belgium


### Particle counting immunoassay (PACIA) V – its application to the determination of human placental lactogen

A. E. Leek
*Technia Diagnostics Ltd., City Road, London, ECV1JX, England.*

F. de Steenwinkel
*St. Antonius hove, Burgermeester Banninglaan 1, Leidschendam, The Netherlands.*

C. L. Cambiaso and P. L. Masson
*Unit of Experimental Medicine, International Institute of Cellular and Molecular Pathology, Université Catholique de Louvain, B–1200 Brussels (Belgium).*

### Introduction

The extent of agglutination of latex particles may be measured with accuracy and precision by counting the residual non-agglutinated particles with a blood cell counter [1]. When the particles are coated with antibodies (Ab), antigens (Ag) can be used as agglutinators. The extent of agglutination will then depend on the concentration of Ag.

This principle is called particle counting immunoassay (PACIA) and has been shown to be suitable for the determination of proteins [1]. However studies of accuracy and precision, as well as comparison with other techniques have not been made. In the present work, it is shown that a clinically useful assay for human placental lactogen (hPL) using PACIA is feasible and offers an alternative to radio- and enzymo-immunoassays.

### Materials and methods

### Antigen and antiserum

Purified hPL for standard solutions and immunisation was supplied by ILS Ltd, (48–50 Bartholomew Close, London EC1 U.K.). Standard solutions were prepared by serial dilution of a concentrated solution of hPL in normal male serum or in 0.1 M glycine-NaOH buffer pH 9, containing 9 g/l NaCl (GBS) and 1 g/l bovine serum albumin (BSA).

Antiserum was raised in a goat. The immunogen was dissolved in 0.9% NaCl, and 1 ml (100 $\mu$g hPL) emulsified with 1 ml of complete Freund's adjuvant was injected intradermally in several sites. The animal was immunised on days 0, 14, 28, 56, 70, 98 and 112, and bled on day 126 for the antiserum used in the tests reported here. IgG was purified from the antiserum by precipitation of other serum proteins with 6, 9-diamino-2-ethoxyacridine lactate (Rivanol). Four volumes of a 0.4% (w/v solution of Rivanol in water were added to 1 volume of antiserum and the glutinous precipitate centrifuged at 5,000 g for 5 min. Excess Rivanol in the supernatant was precipitated by the addition of 125 $\mu$l saturated NaBr per ml of antiserum treated, followed by centrifugation (at 10,000 g for 10 min). The supernatant was dialysed overnight at 4° C against 0.9% NaCl to remove traces of Rivanol. This IgG solution was used for coating latex and for the preparation of F(ab')$_2$ fragments by pepsin digestion as described elsewhere [2].

### Particles

A 50 $\mu$l aliquot of particle suspension (1.1 $\mu$ diameter, 10 % w/w, Dow Chemical Co., Indianapolis, USA) was incubated at room temperature with 1 mg IgG or 100 $\mu$g F(ab')$_2$ in 1 ml of 5-fold diluted GBS (dGBS). For IgG, incubation was for 30 min; then 1 mg of BSA in a small volume of dGBS was added for a further 15 min incubation. For F(ab')$_2$ incubation was for 45 min in the presence of 1 mg BSA. After incubation the latex was washed twice with 1 ml GBS, by centrifugation at 10,000 g for 5 min. After the final wash the latex was resuspended in 2 ml GBS with 1 mg BSA/ml, left for 24 h at 4° C, centrifuged and resuspended in 1 ml GBS with 1 mg BSA/ml. It was then ready for use.

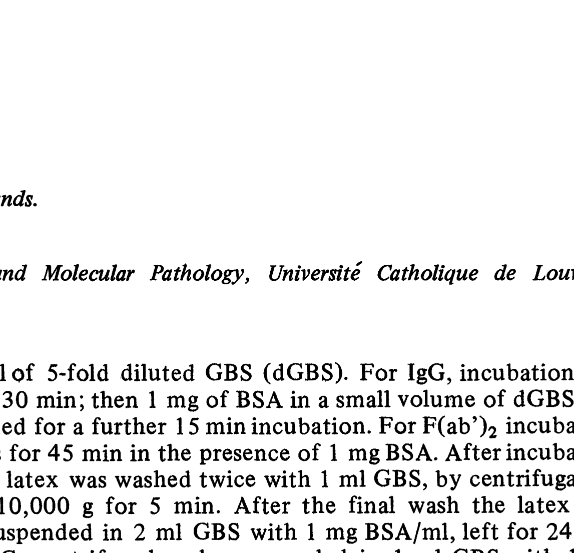

*Figure 1. Flow diagram of manifold (ml/min). All fittings were standard Technicon parts, as follows:-*
*a : PT2; c : 157-0303-02; d : 2 x 190-0051-03; f : 116-0127-02; g : A9; h : A14; i : D1; j : 190-0051-06; k : Ab; l : A9; m : A12; n : D1; o : 190-0051-17; p : all except for the following: b : PT13 fitting modified so that the exit tube along the long axis had an internal diameter of 1.0 mm; e : resample fitting, modified with an extra side-inlet tube for immediate re-bubbling of the reagent stream. Additive was generally GBS containing 10 g BSA/1.*

### Instruments

Particles were counted in the Technicon AutoCounter (Tarrytown, New York, USA) modified electronically so that an upper as well as a lower threshold could be set on the signal from the photomultiplier tube. With the upper threshold, single (unagglutinated, monomer) could be discriminated from multiple (agglutinated, polymer) particles. While this modification is useful in that it increased sensitivity by about 10 %, it is not essential to the basic technique. The electronic device can be obtained from Technicon Instruments, Dublin, Eire.

Tests were at first carried out manually. Aliquots of Ab-coated latex and sample were agitated in 5 ml polystyrene test-tubes on the turntable of a vortex mixer for 20 min, then diluted with 5 ml GBS. Using a Technicon Sampler IV, pump and manifold, aliquots were taken from these tubes, diluted 10- to 40- fold, and passed through the flow cell of the AutoCounter. The final dilution step was calculated to give a maximum of about 3000 particles/sec passing through the flow-cell. The AutoCounter output was continuously traced on a variable-gain 2-pen strip-chart recorder, to record the concentrations of monomer and polymer particles.

Having established that the particle-counting system gave a good reliable response to antigen (hPL) in the samples, the technique was automated using the Technicon continuous-flow system (Figure 1). The manifold was slightly different from that described previously [1]. Samples of serum, suitably prediluted in GBS were aspirated from a Sampler IV at 30 samples/hour with 1:1 sample/wash ratio. The sampler had an extra arm carrying two probes to aspirate Ab-coated latex and another solution (additive) at the same time as the sample. The additive was generally GBS containing 10 g BSA/l to which different reagents, e.g. PEG (polyethylene glycol, mol wt 6000D) or free anti-hPL, were added for specific experiments. The pumping rates were 50 μl/min for sample and latex, and 100 μl/min for the additive. Before the three reagents were mixed in a PT13 fitting, the additive stream was segmented with 100 μl/min of air. The reagent mixture was then pumped through plastic tubing to two incubation coils mounted vertically and in series on a simple vibrator. The vibrating motion was imparted by an induction motor oscillating a spring loaded platform upon which the coils had been mounted. The coils executed a side-to-side motion of 1 mm amplitude at a frequency of 50 Hz. A simply-segmented reagent stream tended to break up during passage through the vibrated coil. By segmenting the "additive" stream before mixing, a number of small air bubbles was introduced between each segmenting bubble, and these stabilised the reagent stream during incubation. The reagent stream, on leaving the incubation coils, was passed through a special resampling fitting, with practically no dead volume. The aliquot removed was immediately re-segmented with air and then twice diluted in series, with GBS containing 0.025 % v/v Tween 20 before being pumped through the AutoCounter flow-cell. The incubation time of the reaction mixture was 24 min, and the total throughput time, 31 min.

### Results

### Manual version

The effect of incubation time was tested using the manual version of PACIA. Increased time markedly enhanced the agglutination and improved the sensitivity (Figure 2). With a 20 min incubation, 0.1 μg/l of hPL could easily be detected. But, as a result of hand pipetting and dilution steps, the coefficient of variation within assay reached 10 %. Accordingly, the automated system was used to obtain all the other results reported.

### Effect of concentration of particles and their antibody loading

Increasing the concentration of particles increased markedly their agglutinability (Figure 3). The same effect was observed as a result of changing the concentration of IgG with which

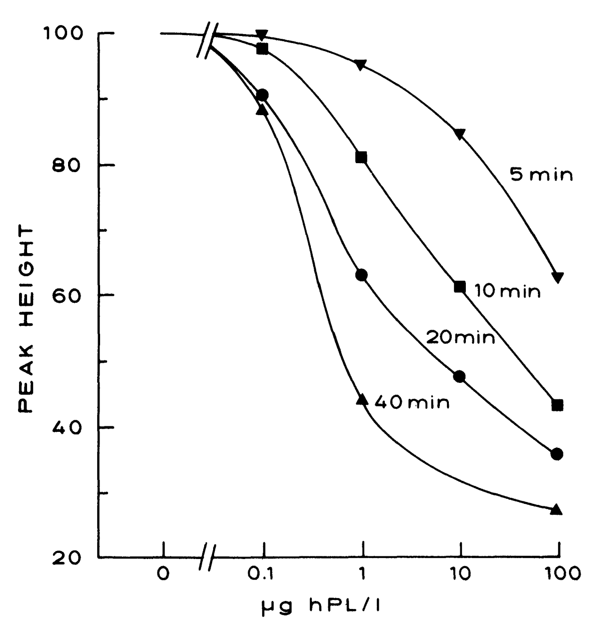

*Figure 2. Effect of incubation time on the agglutination of anti-hPL latex by hPL. 10 μl aliquots of latex reagent and GBS containing 1 g/l BSA and the indicated concentration of hPL were incubated for various times and processed by the manual technique described in Materials and methods. Peak height represents the concentration of particles in arbitrary units.*

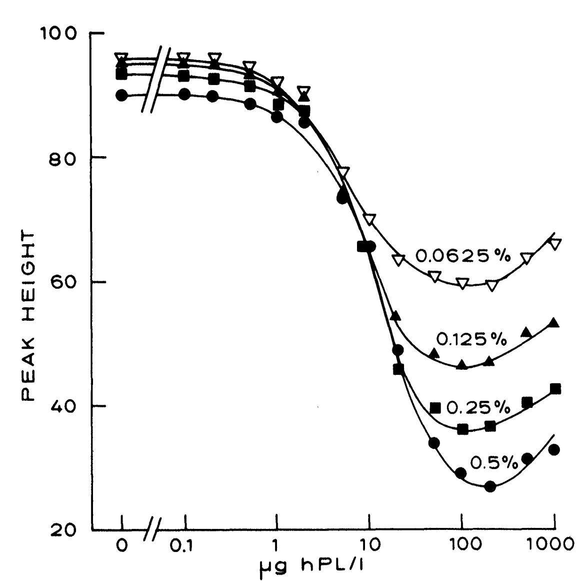

*Figure 3. Effect of latex concentration on its agglutination by hPL. The determination was carried out with the automated system, aspirating samples of GBS containing 1 mg BSA/ml and the indicated concentration of hPL, latex at a concentration of 0.5 %, 0.25 %, 0.125 % or 0.0625 % (w/v), and an additive of GBS with 10 g BSA/l. Particle concentration is expressed as chart peak height, adjusted for maximal response for each concentration of latex.*

the particles were prepared, i.e. the amount absorbed onto their surfaces (Figure 4). The relationship between IgG concentration and surface absorption has been described elsewhere [3].

**Sensitivity**

Polymers such as dextran or PEG enhance immunological agglutination or precipitation [4]. The sensitivity of the assay of hPL by PACIA in the presence of PEG was clearly increased (Figure 5). Without PEG, 5 μg/l of hPL could barely be detected, whereas in the presence of 20 g/l of PEG, 5 μg/l of hPL decreased the number of particles by 75 %. According to Hellsing [4], this effect of PEG could be explained in terms of a steric exclusion of the AgAb complexes from the domain of the polymer. It must be noted that PEG is not effective in all applications of PACIA (in preparation).

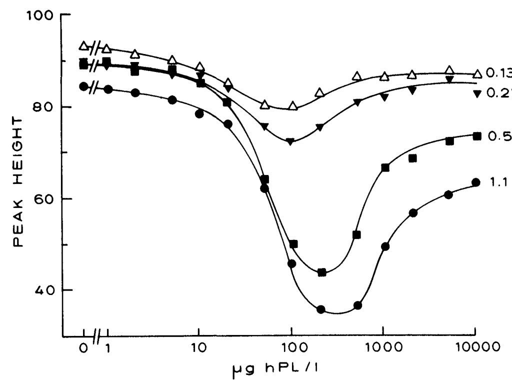

*Figure 4. Effect of different anti-hPL IgG loadings of latex on its agglutination by hPL. Conditions as for Figure 3, but using different latex preparations prepared as described in Materials and methods, incubation being at the various IgG concentrations indicated.*

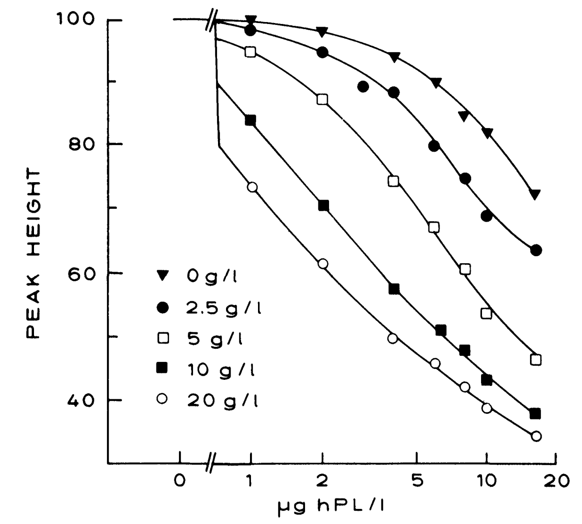

*Figure 5. Effect of PEG6000 on the agglutination of anti-hPL latex reagent by hPL measured with the automated system. Serum samples with indicated hPL concentrations diluted 1:200 with GBS, were aspirated with latex and an additive of GBS containing 1 g BSA/l and PEG for final concentration as indicated.*

The sensitivity of the system could also be increased by using the ratio of the concentrations of unagglutinated (monomer) to agglutinated (polymer) particles, instead of that of monomers alone (Figure 6). The initial slope of the standard curve obtained with this ratio was also steeper. In the presence of low concentrations of agglutinator there is a small reduction in the large number of monomers, but a proportionately larger increase in the small number of polymers. Hence, the use of the ratio will give a greater response to a given concentration of Ag than will the use of the monomer concentration, and will also give a more accurate response than that of polymer concentration where only a few particles are counted.

**Table 1. Reduction of the latex count by various rheumatoid sera as the percentage of the number of particles before addition of the agglutinator**

| | Serum from patients named | | | |
|---|---|---|---|---|
| | Gui | Juv | Ler | Mae |
| IgG-coated latex | 68 | 53 | 82 | 32 |
| F(ab')$_2$-coated latex | 2 | 1.5 | 0.5 | 2.5 |

**Table 2 Inter-assay precision of hPL standard curve measurement**

| hPL concentration | Peak height or particle concentration in arbitrary units | | |
|---|---|---|---|
| | Mean | Standard deviation | Coefficient of variation |
| (mg/l) | | | |
| 1 | 92.1 | 1.5 | 1.63 % |
| 2 | 80.5 | 1.55 | 1.93 % |
| 4 | 59.0 | 2.15 | 3.64 % |
| 6 | 42.5 | 2.4 | 5.65 % |
| 8 | 32.4 | 2.0 | 6.17 % |
| 10 | 27.4 | 1.35 | 4.93 % |
| 16 | 21.2 | 0.6 | 2.83 % |

Assays were run on the PACIA automated system on 5 successive days.

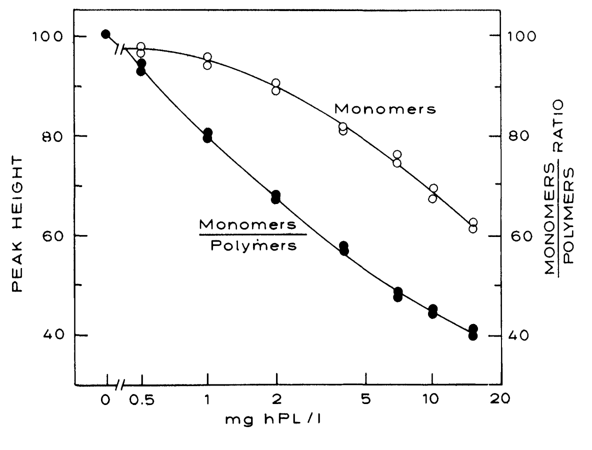

*Figure 6. Standard curves from automated PACIA assay. Serum samples containing indicated concentrations of hPL diluted 1:400 with GBS were aspirated with latex, and GBS (50 g BSA/l). Results expressed in concentration of monomer particles, or the ratio of monomer to polymer particle concentrations.*

### Accuracy and precision

When the agglutination reaction was carried out in serum several effects were observed. Normal sera, in general, caused a decrease in specific agglutination and in the sensitivity of the reaction (Figure 7). There was also a nonspecific agglutination of the Ab-coated latex (Figure 7). These effects were much less marked with latex coated with the $F(ab')_2$ fragments of the goat IgG anti-hPL. This $F(ab')_2$-latex also prevented the agglutination by rheumatoid factor. Four rheumatoid sera diluted 1/200 in GBS strongly agglutinated IgG-latex, but not the $F(ab')_2$-latex (Table 1).

Thirty-two serum samples from an obstetric clinic were assayed by PACIA, and the results compared with those obtained with "Phadebas" RIA. Initially, either IgG-latex with sera diluted 1/1000, or $F(ab')_2$-latex with sera diluted 1/250 were used. However, by adding free anti-hPL to the reagent stream it was possible to decrease the sensitivity of the reaction sufficiently to use a more practical dilution of 1/10 with no loss of precision or accuracy. The correlation between the results by PACIA and by radioimmunoassay using the 1/250 dilution is shown in Figure 8; similar results were obtained using the other dilutions, i.e. 1/1000 and 1/10.

The precision of the fully automated PACIA was tested by running hPL standard curves with two batches of IgG-latex each day for a week (Table 2). The inter-assay coefficient of variation for each standard concentration was less than 6.5 %.

### Discussion

The present results confirmed the potential of PACIA for the determination of proteins. The sensitivity of PACIA as applied to hPL is impressive. With a 20 min incubation this method can measure concentrations that require an incubation of 20 hours in radioimmunoassay. In the best conditions of PACIA, 0.1 $\mu$g/l of hPL (4.5 x $10^{-12}$M) reduced the number of particles by 10 %. The latex concentration being 0.86 x $10^{12}$ particles/l, 54 molecules were necessary to agglutinate 2 particles.

Serum interference was discussed in detail previously [2]. Non-specific agglutination is mainly due to rheumatoid factor, which is found in many sera when a sensitive agglutination technique is used. The use of $F(ab')_2$ fragment prevents this type of interference and, to some extent, the agglutination inhibition produced by some sera. It is suspected that the inhibitory factor is IgG rheumatoid factor which, by coating the particles, masks the antibodies and hence decreases the agglutinability of the particles.

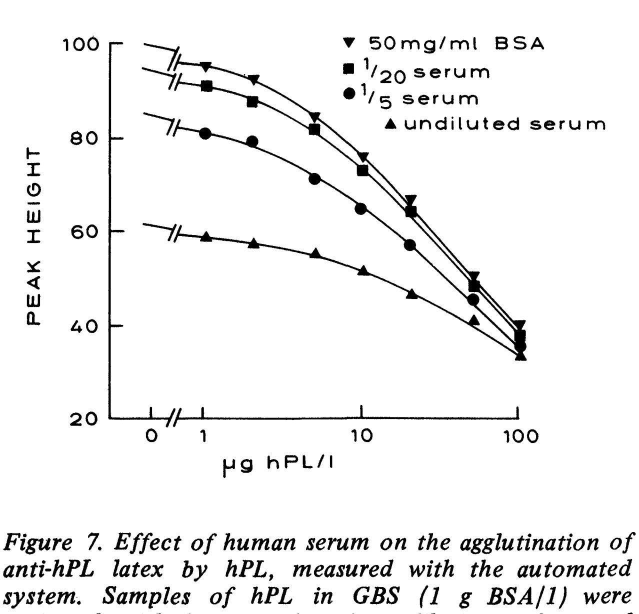

*Figure 7. Effect of human serum on the agglutination of anti-hPL latex by hPL, measured with the automated system. Samples of hPL in GBS (1 g BSA/l) were aspirated with latex, and various dilutions of normal human serum or GBS containing 50 g/l BSA.*

As a practical assay system PACIA has both unrealised potential and advantages over other techniques. It has adequate sensitivity for the determination of most proteins of biological and clinical interest and seems to be more rapid than most other techniques. PACIA also has the advantages of not using radioisotopes and of allowing easy automation. Applications of PACIA to the determination of rat IgA idiotypes [5], mouse anti-IgG autoantibodies [6], and circulating immune complexes [3,7,8] have been reported. The agglutination has been measured with a Coulter counter model ZB [8].

ACKNOWLEDGEMENT

This work was supported by Technicon Instruments Corporation (Tarrytown, New York). The authors are grateful to Dr. H. Holy and C. Richard for helpful discussion and correction of the manuscript and would like to thank Dr. K. Thomas for supplying some of the serum samples.

REFERENCES

[1] Cambiaso, C. L., Leek, A. E., De Steenwinkel, F., Billen, J. and Masson, P. L., *Journal of Immunological Methods*, 1977, **18**, 33.

[2] Limet, J. N., Moussebois, C. H., Cambiaso, C. L., Vaerman, J. P. and Masson, P. L., *Journal of Immunological Methods*, 1979, **28**, 25.

[3] Cambiaso, C. L., Riccomi, H., Sindic, C. and Masson, P. L., *Journal of Immunological Methods*, 1978, **23**, 29.

[4] Hellsing, K., *Immunochemistry*, 1972, **9**, 753.

[5] Jackson, G. D. F., Lemaître-Coelho, I., Vaerman, J. P., Bazin, H., and Beckers, A., *European Journal of Immunology*, 1978, **8**, 123.

[6] Van Snick, J. L., and Masson, P. L., *Journal of Experimental Medicine*, 1979, **149**, 1519.

[7] Cambiaso, C. L., Sindic, C., and Masson, P. L., *Journal of Immunological Methods*, 1979, **28**, 13.

[8] Levinsky, R. J., and Soothill, J. F., *Clinical and Experimental Immunology*, 1977, **29**, 428.

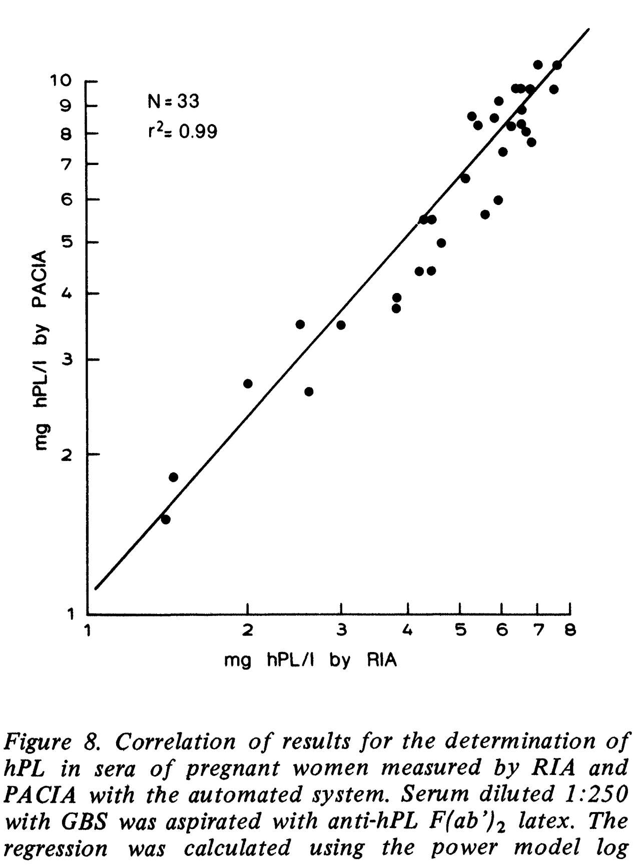

*Figure 8. Correlation of results for the determination of hPL in sera of pregnant women measured by RIA and PACIA with the automated system. Serum diluted 1:250 with GBS was aspirated with anti-hPL $F(ab')_2$ latex. The regression was calculated using the power model log $y = b \log x + \log a$.*

